# Concomitant glenohumeral injuries in patients with distal clavicle fractures undergoing arthroscopic-assisted surgery: a systematic review

**DOI:** 10.1186/s13018-022-02919-7

**Published:** 2022-01-15

**Authors:** Theodorakys Marín Fermín, Filippo Migliorini, Emmanuel Papakostas, Khalid Al-Khelaifi, David Ricardo Maldonado, Jean Michel Hovsepian, Nicola Maffulli

**Affiliations:** 1 Aspetar Orthopedic and Sports Medicine Hospital, Doha, Qatar; 2 Department of Orthopedic, Trauma, and Reconstructive Surgery, RWTH Aachen University Hospital, Pauwelsstr. 30, 52074 Aachen, Germany; 3 Kerlan-Jobe Institute, Los Angeles, CA USA; 4 Department of Sports Orthopedics, Hessing Klinik, Augsburg, Germany; 5 Department of Medicine, Surgery and Dentistry, University of Salerno, Baronissi, Italy; 6 School of Pharmacy and Bioengineering, Keele University School of Medicine, Stoke on Trent, England; 7 Barts and the London School of Medicine and Dentistry, Centre for Sports and Exercise Medicine, Mile End Hospital, Queen Mary University of London, London, England

**Keywords:** Glenohumeral, Distal clavicle fractures, Arthroscopy

## Abstract

**Background:**

To determine the incidence of concomitant intra-articular glenohumeral injuries in patients undergoing surgical management from distal clavicle fractures (DCF) with shoulder arthroscopy and their impact on outcome.

**Methods:**

This systematic review was conducted following the PRISMA guidelines. PubMed, EMBASE, and Virtual Health Library databases were accessed in October 2021. All the clinical studies evaluating the surgical management of DCF and using concomitant intra-operatory shoulder arthroscopy were included. Studies that did not specify the concomitant injury type were not eligible. Data from the incidence of intra-articular glenohumeral injuries, injury type, length of the follow-up, and clinical outcomes were retrieved. The quantitative content assessment was performed using the STROBE statement checklist. Evaluation of the publication bias of the included studies was performed using the risk of bias assessment tool for systematic reviews.

**Results:**

Data from five retrospective and five prospective cohort studies were analyzed. Eight of the included studies were conducted on patient cohorts with Neer type II injuries. Data pooling revealed a mean of 17.70% of concomitant glenohumeral injuries, whereas 84.21% of them required additional surgical management (Table 1). Rotator cuff injuries, labral tears, and biceps pulley lesions were the most common concomitant injuries.

**Conclusion:**

Preoperative MRI or diagnostic arthroscopy to evaluate glenohumeral associated injuries to DCF should be recommended.

## Introduction

Clavicle fractures account approximately 2.6–4% of all fractures in the adult population [1, 2]. Of them, distal clavicle fractures (DCF) account up to 28% [1, 2]. The majority of DCF occur after a direct fall over the shoulder or, in smaller part, after a fall on outstretched hand [3–6]. Management of DCF can be challenging. Most classifications for DCF are mainly based on the configurations of bone fragments (stable or unstable) and the location in relation to the coracoclavicular ligaments [3, 7–10]. Stable lesions can be treated conservatively; however, failing to identify unstable lesions could result in pseudoarthrosis/nonunion and poor shoulder function [11–14]. Several surgical techniques have been described to manage unstable DCF, but to the best of our knowledge, no consensus has been reached [11, 15–18]. Surgical management can be categorized as rigid (locking and hook plates) and elastic (Kirshner-wire fixation, tension band wiring, suture anchors, button suture systems) fracture fixation, or a combination of both. The surgical procedure can be open, arthroscopically assisted, or fully arthroscopic [17, 19].

The incidence of associated lesion after DCF is highly variable [6, 20–24]. Preoperative physical examination to investigate concomitant injuries to DCF can be difficult because of pain and inflammation. Moreover, MRI or diagnostic arthroscopy of the glenohumeral joint to investigate associated is not routinely performed [24]. This systematic review investigated the incidence of concomitant intra-articular glenohumeral injuries in patients undergoing surgical management of DCF using concomitant intra-operative shoulder arthroscopy.

## Methods

### Search strategy

This systematic review was conducted following the Preferred Reporting Items for Systematic Reviews and Meta-Analyses (PRISMA) guidelines [25]. Two independent reviewers (T.M.F., J.M.H.) accessed PubMed, EMBASE, and Virtual Health Library databases in October 2021. The following terms "distal clavicle fracture" and "arthroscopy” were used alone and in combination with the Boolean operators AND and OR. Inclusion and exclusion criteria were established before the search and were used to identify potentially eligible studies by title and abstract screening. Disagreements between reviewers were resolved by a third investigator (E.P.). The bibliography of the included studies was screened by hand to identify additional studies.

### Eligibility criteria

All the clinical studies evaluating the surgical management of DCF and using concomitant intra-operatory shoulder arthroscopy were included. Only studies in English were included. Only studies published in peer reviewed journal with a minimum of 5 patients were considered. Reviews, comments, opinions, and editorials were not eligible. Studies which reported data on insolated DCF without arthroscopy were not eligible. Studies which did not specify the concomitant injury type were also not eligible. Studies which reported shoulder injuries associated with DCF in other forms rather than a direct arthroscopic visualization were not included.

### Data extraction

Two independent investigators (T.M.F., J.M.H) performed data extraction. Studies generalities (author, year, type of study, and level of evidence) were extracted. Data from the following endpoints were retrieved: number of patients, classification, incidence of intra-articular glenohumeral injuries, injury type, length of the follow-up, clinical outcomes.

### Methodological quality assessment

The quantitative content assessment was performed using the Strengthening the Reporting of Observational Studies in Epidemiology: the STROBE statement checklist (SSc) [26].

### Assessment of publication bias

Evaluation of the publication bias of the included studies was performed using the risk of bias assessment tool for systematic reviews (ROBIS) [27]. This tool was developed to assess the risk of bias in systematic reviews and meta-analysis. The ROBIS is composed by three parts: (1) assessment of relevance (optional), (2) identification of concerns with the review process (study eligibility criteria; identification and selection of studies; data collection and study appraisal; and synthesis and findings), and (3) evaluation of the risk of bias in the review process, results and conclusions.

### Statistical analysis

The statistical analysis was performed using IBM SPSS Version 19 and Microsoft Excel 2016 (Microsoft, USA). Data were presented in tables using absolute values, standard deviations, and percentages from individual studies. Values of *P* < 0.05 were considered statistically significant.

## Results

### Search results

**Fig. 1:**
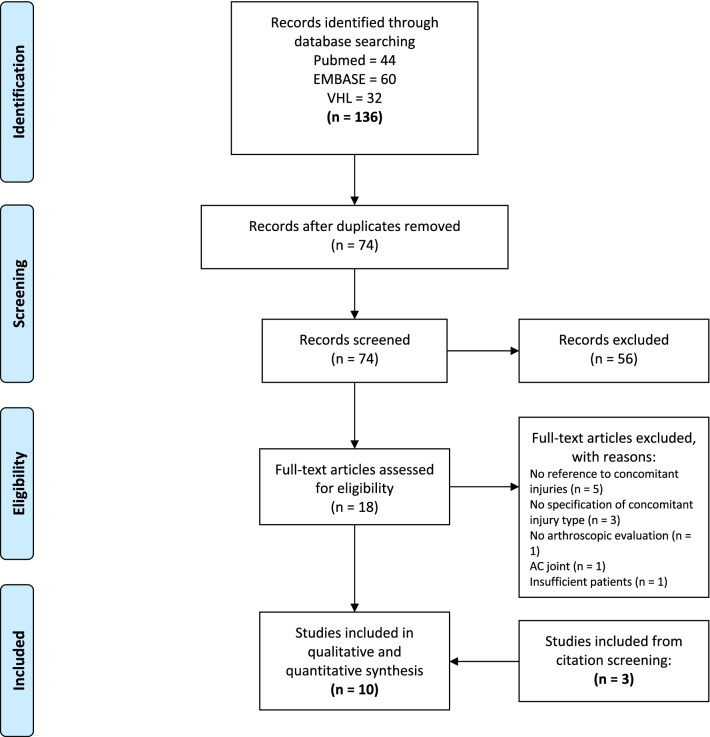
Flow chart of the literature search

The initial literature search yielded 74 potentially relevant records after the removal of duplicates (N = 27). Titles and abstracts were screened, and 18 articles for full-text evaluation were retrieved. Seven studies met the predetermined eligibility criteria [21–24, 28–30], and three additional studies were included after citation screening [31–33] (Fig. 1). There were five retrospective [21, 24, 28, 31, 32] and five prospective cohort studies [22, 23, 29, 30, 33].

### Methodological quality assessment

The SSc was used to assess the quality of individual studies in the present investigation (Table 1). The average SSc value was 26.30 of 32 (range 22–31), indicating a good quality of the methodological assessment.

**Table 1:** STROBE Statement checklist score of included cohort studies

Study	Years	Level of evidence	Score (max. 32)
Dey Hazra et al. [31]	2020	IV	29
Helfen et al. [24]	2018	IV	31
Kuner et al. [32]	2018	IV	26
Sautet et al. [21]	2018	IV	25
Xiong et al. [29]	2018	IV	29
Blake et al. [22]	2017	IV	22
Cisneros and Reiriz [28]	2017	IV	25
Beirer et al. [23]	2015	IV	26
Kraus et al. [33]	2015	IV	27
Loriaut et al. [30]	2013	IV	23

### Assessment of publication bias

**Fig. 2:**
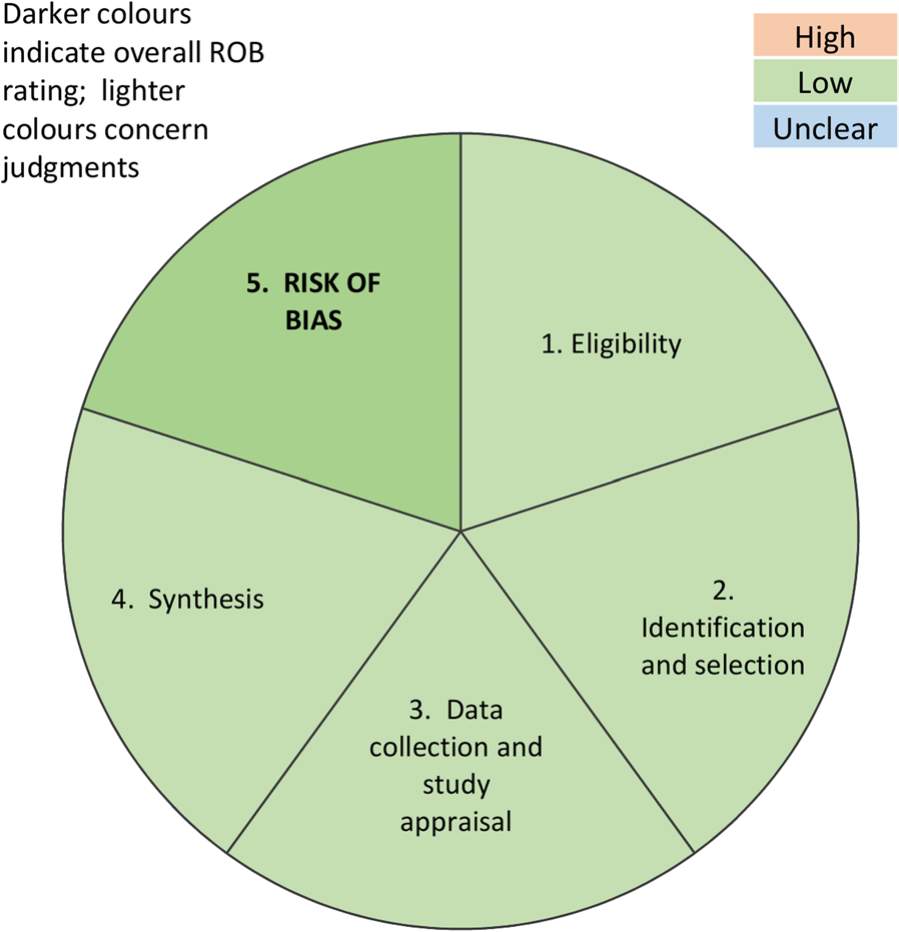
Assessment of publication bias

The risk of bias in the review was low (Fig. 2). A low heterogeneity among the included studies was observed in the arthroscopic assessment of intra-articular glenohumeral concomitant injuries, in the standardization of the surgical procedure, and postoperative management. Most studies clearly defined the type of lesion and referred to standardized classifications.

### Synthesis of Results

Eight of the included studies were conducted on patient cohorts with Neer type II injuries [21, 22, 24, 28–30, 32, 33]. Data pooling revealed a mean of 17.70% of concomitant glenohumeral injuries, whereas 84.21% of them required additional surgical management (Table 2).

**Table 2:** Incidence of intra-articular injuries in distal clavicular fractures and injury type among the included studies

Study	Number of patients	Fracture classification	Incidence of intra-articular injuries	Injury type	Follow-up (mean)	Outcomes
Dey Hazra et al. [31] 2020 Retrospective cohort study	8	Jäger and Breitner IIA/Neer IIB	37.5% (3 patients)	Labral tear (1) SLAP lesion (1) Pulley lesion (1) Biceps tendon lesion (1) PASTA – Ellman A1 (1) SSC partial rupture – Fox and Romeo 2 (1) SGHL injury (1)	36 (36.6 ± 14.3) months	Outcome differences were not evaluated Additional surgical treatment was required in patients with concomitant injuries
Helfen et al. [24] 2018 Retrospective cohort study	41	Neer type II	27% (11 patients)	SLAP lesion (1) SSP transmural tears (3) SSP partial ruptures (5) SSC partial rupture (1) Pulley lesion (1) Bankart lesions (2)	12 months	No outcome differences in Constant score and Oxford shoulder score were found regarding concomitant injuries Additional surgical treatment, other than debridement, was required in 5 patients with concomitant injuries Out of 11 patients with concomitant glenohumeral injuries, five of them were diagnosed during the primary arthroscopy, and six of them during the diagnostic arthroscopy at the time of hardware removal In the subgroup of existing concomitant injuries, out of all measured functional outcome parameters implant removal and late arthroscopy benefitted patients' functional outcomes
Kuner et al. [32] 2018 Retrospective cohort study	20	Neer type II	0%	None	12–50 (18.7) months	
Sautet et al. [21] 2018 Retrospective cohort study	14	Neer type IIb	0%	None	6–55 (20) months	
Xiong et al. [29] 2018 Prospective cohort study	28	Neer type II	14.29% (4 patients)	Bankart lesion (1) Rotator cuff injury (1) Glenolabral articular disruption (1) Acromioclavicular joint arthritis (1)	7–160 (57) months	Concomitant injuries were repaired arthroscopically at the time of fracture fixation Rehabilitation time was lengthened in patients with concomitant injuries Outcome differences were not evaluated
Blake et al. [22] 2017 Prospective cohort study	17	Neer type II	0%	None	The mean duration from surgery to the most recent follow-up was 12 months	
Cisneros and Reiriz [28] 2017 Retrospective cohort study	9	Neer type IIb	22.22% (2 patients)	Rotator cuff tears (2)	46–52 (49) months	Concomitant injuries were repaired when detected Outcome differences were not evaluated
Beirer et al. [23] 2015 Prospective cohort study	28	Jäger and Breitner I, II, and III	46% (13 patients)	SLAP (4) Pulley lesions – Habermeyer III (3) PASTA (1) SSC lesion – Fox and Romeo II (1)		Additional surgical treatment was required in 8 of 13 (61.54%) patients with concomitant injuries Outcome differences were not evaluated
Kraus et al. [33] 2015 Prospective cohort study	20	Neer type II	10% (2 patients)	SSC tear – Fox and Romero I and II (2)	13–38 (23) months	Patients with concomitant injuries required surgical treatment Outcome differences were not evaluated
Loriaut et al. [30] 2013 Prospective cohort study	24	Neer type IIb	8.33% (2 patients)	Rotator cuff injury (1) Labral tear (1)	24–51 (35) months	Patients with concomitant injuries required surgical repair Outcome differences were not evaluated
Total	209		17.70%			

Helfen et al. [24] assessed the clinical outcomes in patients with and without concomitant injuries, finding no differences in Constant and Oxford shoulder score at last follow-up. Xiong et al. [29] reported a prolonged rehabilitation in patients with concomitant injuries. Concomitant glenohumeral injuries were summarized (Table 3).

**Table 3:** Distribution of concomitant injuries according to their type in distal clavicular fractures among the included studies

Injury type (number of injuries)	%
**ROTATOR CUFF INJURY (19)**	**50.00**
SSP partial ruptures (5)	26.32
SSC tears (5)	26.32
Non-specified (4)	21.05
SSP transmural tears (3)	15.79
PASTA (2)	10.53
**LABRAL TEAR (12)**	**31.58**
SLAP lesion (6)	50
Bankart lesions (3)	25
Non-specified (2)	16.67
Glenolabral articular disruption (1)	8.33
**PULLEY LESION (5)**	**13.16**
**OTHER INJURIES (2)**	**5.26**
Biceps tendon lesion (1)	
SGHL injury (1)	

The bold values correspond to the total of those types of injuries

PASTA: partial articular supraspinatus tendon avulsion; SGHL: superior glenohumeral ligament; SLAP: superior labrum anterior–posterior; SSC: subscapularis; SSP: supraspinatus

## Discussion

The present systematic review highlighted that 17.70% of patients with acute DCF evidenced concomitant glenohumeral injuries. Rotator cuff injuries, labral tears, and biceps pulley lesions were the most common concomitant injuries, requiring additional surgical treatment in 84.21% of cases. This incidence is similar to those reported following after acromioclavicular dislocations [20]. This similarity may result from to the similar mechanism of injury [6]. Preoperative MRI or diagnostic arthroscopy to evaluate glenohumeral associated injuries to DCF should be recommended.

The management of concomitant injuries to the DFC have demonstrated clinical improvement and may avoid persistent symptoms and early onset of degenerative changes [34–36]. However, the current evidence is not strong enough to ascertain whether concomitant glenohumeral injuries in DCF may affect the final outcome of management of these injuries.

DCF have been traditionally managed through open approaches with very satisfying outcomes, and further imaging or arthroscopic assessments are related to increased surgical time and costs [28, 37]. However, the acute pain following an acute DCF, or the administration of pain medications, may jeopardize the presence of concomitant shoulder injuries. Therefore, the presence of concomitant injuries should be evaluated using preoperative MRI or diagnostic arthroscopy in patients with DCF. Whether to combine the management of DCF with a simultaneous or delayed additional glenohumeral intervention should be evaluated for each patient, and surgery should be individualized.

This study has several limitations. The small number of included studies and relatively small sample size is the most important limitation of the present systematic review. The retrospective nature of 50% (5 of 10) of included studies increases the risk of selection bias. None of the included studies performed randomization or blinding, thus increasing the risk of detection bias. Most of the included studies were conducted on patients with DCF type II according to the Neer [7]. Thus, results from this systematic review may be not fully generalized. Further high-quality investigations should be performed to overcome current limitations and to evaluate the efficacy and safety of simultaneous glenohumeral interventions.

## Conclusion

17.70% of patients with a DCF evidenced concomitant glenohumeral injuries. Rotator cuff injuries, labral tears, and biceps pulley lesions were the most common concomitant injuries, requiring additional surgical treatment in 84.21% of cases. Preoperative MRI or diagnostic arthroscopy to evaluate glenohumeral associated injuries to DCF should be recommended.

## Data Availability

The datasets generated during and/or analyzed during the current study are available throughout the manuscript.

## Abbreviations

SSc: STROBE statement checklist
ROBIS: Risk of bias assessment tool for systematic reviews
PASTA: Partial articular supraspinatus tendon avulsion
SGHL: Superior glenohumeral ligament
SLAP: Superior labrum anterior–posterior
SSC: Subscapularis
SSP: Supraspinatus
